# Barriers to using physical assessment skills in clinical practices among nursing students: a cross-sectional study

**DOI:** 10.3389/fmed.2026.1799059

**Published:** 2026-03-23

**Authors:** Fatma Abdou Eltaib, Ibrahim Naif Alenezi, Fathia Ahmed Mersal, Ohoud Naif Aldughmi, Taghreed Hussien Aboelola, Lobna Mohamed Mohamed Abu Negm

**Affiliations:** 1 Medical-Surgical Nursing Department, Faculty of Nursing, Northern Border University, Arar, Saudi Arabia; 2 Public Health Nursing, Faculty of Nursing, Northern Border University, Arar, Saudi Arabia; 3 Emergency & Critical Care Nursing Department, Faculty of Nursing, Northern Border University, Arar, Saudi Arabia

**Keywords:** barriers, clinical practice, nursing students, physical assessment skills, Saudi Arabia

## Abstract

**Purpose:**

To explore the barriers to physical assessment skills among nursing students at Northern Border University.

**Methods:**

A descriptive cross-sectional study with a sample size of 260 participants was conducted. A self-administered online questionnaire comprising demographic data for nursing students and barriers to physical assessment skills was used. The survey used a five-point Likert scale with responses ranging from “strongly disagree” to “strongly agree” and scores from 1 to 5 points.

**Results:**

Findings revealed that 68.5% were aged 20–29, 67.7% were female, and 75.8% were single. Most were in their third year (56.5%) and had a GPA above 3.5 (80.8%). Many believe physical assessment is mainly a physician’s role. The most prominent barriers reported by respondents included reliance on technology, lack of time, and ward culture. Females reported more barriers, mainly due to technology reliance and low confidence (*p* < 0.05). Regular program students faced more challenges than those in bridging programs (*p* < 0.05) Program type, age, and gender significantly predicted perceived barriers (*p* < 0.05).

**Conclusion:**

The current study findings indicate significant barriers related to gender, age, and program type, with female students reporting greater challenges. The research emphasizes the need for targeted educational interventions, such as structured simulation and clinical mentorship, to enhance nursing students’ competence in physical assessments.

## Introduction

Physical and health assessment skills are foundational competencies in undergraduate nursing education, serving as critical determinants of nurses’ preparedness to manage complex patient interactions (1, 2). A comprehensive physical examination involves systematic data collection through inspection, palpation, percussion, and auscultation, supplemented by objective measurements such as vital signs, height, weight, and oxygen saturation (3). As one of the three pillars of diagnostic evaluation, physical examination enables healthcare providers, including nurses, to validate clinical hypotheses derived from patient histories, track disease progression, and inform care planning (4, 5). Despite its clinical importance, research indicates that nurses and nursing students underutilize physical assessment skills in practice, with only 11–29% of techniques taught in curricula routinely applied in clinical settings (6, 7). This discrepancy highlights the ongoing challenges in effectively translating theoretical knowledge into clinical proficiency, particularly within the complex and demanding environments of modern healthcare. Addressing these gaps is especially relevant under the transformative goals of Saudi Vision 2030, which prioritizes the development of a highly skilled and competent healthcare workforce to ensure superior patient safety and care quality.

Nursing education often relies on traditional lecture-based methods, creating a disconnect between classroom instruction and clinical realities (8). For example, Saudi Arabian nursing students report that overcrowded curricula and insufficient hands-on training leave them unprepared to apply theoretical knowledge during clinical rotations (9). This misalignment is compounded by curricula that prioritize biomedical-model frameworks, focusing on disease-specific assessments rather than holistic patient evaluation (7). Often overburdened by administrative tasks, clinical instructors lack time to reinforce physical assessment skills, further widening the theory–practice gap (10).

Self-confidence and clinical judgment are pivotal for practical skill application. Studies reveal that nursing students frequently avoid physical assessments due to fear of inaccuracies or misinterpretations (11). At Saudi institutions like Majmaah University, 42–50% of students lack confidence in performing critical techniques such as auscultation or palpation, attributing this to limited practice opportunities and inadequate feedback during clinical placements (9). Competence is further undermined by reliance on technology (e.g., electronic monitoring systems), which reduces hands-on assessment frequency and perpetuates skill decay (12).

Ward culture and interdisciplinary dynamics significantly influence skill utilization. In Saudi clinical settings, students face discouragement from healthcare staff who perceive physical assessments as physician-exclusive tasks (13). Time constraints, high nurse-to-patient ratios, and frequent interruptions also hinder thorough assessments, with over 60% of students citing these as primary barriers (9). Equipment shortages, such as limited access to stethoscopes or otoscopes, and inconsistent role modelling by senior nurses exacerbate the problem (14).

The Northern Border Province exemplifies a critical context for this issue. Despite Saudi Arabia’s Vision 2030 goals to enhance healthcare quality, nursing education in the region faces persistent challenges, including faculty shortages and outdated training facilities (13, 15). Studies in Riyadh and regional hospitals reveal that 67% of nursing students perceive clinical training as inadequately aligned with real-world demands, resulting in gaps in patient safety and care quality (9). These disparities are particularly pronounced in rural areas, where limited access to simulation labs and mentorship opportunities stifle skill development (16).

This study addresses a critical gap by examining barriers among nursing students at Northern Border University, a population underrepresented in existing literature. By identifying context-specific challenges, the findings will inform strategies to optimize clinical training, enhance competency-based education, and ultimately improve patient outcomes in Saudi Arabia’s evolving healthcare landscape (17). Therefore, conducting a comprehensive analysis of the barriers nursing students face in performing physical assessments is important, as it serves as the basis for this research. By understanding the barriers to physical assessment among nursing students, we can develop a more thorough approach to enhancing quality patient care in clinical settings.

## Materials and methods

### Study design

A descriptive cross-sectional study was conducted to assess barriers to physical assessment skills among nursing students. This design was chosen to estimate prevalence and explore associations between barriers and student characteristics at a single time point, supporting efficient multivariable analysis and informing targeted educational interventions (18). Reporting followed the STROBE guidelines.

### Setting

The study took place at the Faculty of Nursing, Northern Border University (NBU), Arar, Saudi Arabia, from February 5 to April 30, 2025. NBU offers a 4-year Bachelor of Science in Nursing (regular program) and a bridging track for diploma-qualified nurses. Clinical training occurs across affiliated hospitals and primary care centers, with approximately 1,200 clinical hours over 4 years for regular students and 600 h for bridging students, providing diverse exposure to medical-surgical, critical care, pediatrics, maternity, psychiatric, and community health contexts.

### Target population and sampling

*Population*. All NBU nursing students enrolled in the 2024–2025 academic year who had completed the Health Assessment course and commenced clinical training were eligible. Two cohorts were included: regular undergraduates (*N*₁ = 240) and bridging students (*N*₂ = 96), forming an accessible population of *N* = 336.

*Sampling*. A stratified random sampling strategy by program type (regular vs. bridging) ensured proportional representation and enabled subgroup comparisons while mitigating selection bias. Within strata, simple random sampling was executed using SPSS random case selection with a documented seed to ensure reproducibility. Invitations were distributed via institutional email with up to three weekly reminders.

### Sample size

Sample size was derived using the finite population proportion formula for cross-sectional studies:

$$n=\frac{Z^{2}p(1-p)N}{E^{2}(N-1)+Z^{2}p(1-p)}$$

*Parameters*: *Z* = 1.96, *p* = 0.5, *E* = 0.05, with finite population correction. Target sizes were *n*₁ = 78 (bridging) and *n*₂ = 182 (regular), yielding a total *n* = 260 (77.4% of the population). Post-hoc precision estimates showed margins of error below 5% for strata and overall. Power analysis (G*Power 3.1.9.7) indicated >80% power to detect medium effects (Cohen’s *d* ≥ 0.5) at *α* = 0.05.

### Eligibility criteria

*Inclusion*. (1) Current NBU enrollment during the study period; (2) completion of Health Assessment (NUR 301, regular; NUR 401, bridging) with ≥C; (3) ≥ 120 h of supervised clinical practice; (4) informed consent; and (5) Arabic language proficiency.

*Exclusion*. (1) Students without Health Assessment or clinical initiation; (2) students on leave/suspension; (3) acute physical/psychological conditions compromising response quality (self-disclosed); and (4) incomplete submissions (<80% of the 38 barrier items).

### Instrument

#### Source and structure

Barriers to physical assessment were measured using an Arabic-adapted version of the 38-item Barriers to Physical Assessment Scale, originally developed by (19) and subsequently refined (20). Items are organized across seven theoretically grounded subscales informed by social cognitive theory and situated learning frameworks: (i) Reliance on Others/Technology; (ii) Lack of Time/Interruptions; (iii) Ward Culture; (iv) Lack of Confidence; (v) Lack of Nursing Role Models; (vi) Lack of Influence on Patient Care; and (vii) Specialty-Specific Challenges. Responses were recorded on a 5-point Likert scale (1 = *strongly disagree* to 5 = *strongly agree*).

*Scoring Procedure and Reverse Coding*. Before computing subscale and total scores, all positively worded items, including those reflecting perceived support, availability of role models, and recognized clinical value of physical assessment, were identified and systematically reverse-scored (i.e., a response of 5 was recoded as 1, 4 as 2, 3 remained 3, 2 as 4, and 1 as 5). This recoding procedure ensured that all items were directionally consistent, such that higher scores uniformly reflect greater perceived barriers to physical assessment. Subscale scores were derived by summation of constituent items following reverse coding; total scores ranged from 38 to 190, with higher scores consistently indicating greater levels of perceived barriers. This scoring convention was applied uniformly across the pilot and main samples prior to all psychometric analyses.

#### Cultural adaptation and pilot testing

Cross-cultural adaptation of the scale followed the rigorous five-step protocol proposed by Beaton et al. (21), comprising forward translation, synthesis, back-translation, expert committee review, and cognitive debriefing, to establish semantic, idiomatic, experiential, and conceptual equivalence between the original and adapted versions. The multidisciplinary expert committee comprised nursing faculty, clinical nurse educators, a psychometrician, a certified medical translator, and a cultural anthropologist with expertise in Gulf Cooperation Council (GCC) health systems.

Cultural refinements specifically addressed gender-segregated care environments, prevailing norms of modesty and patient privacy, family-centered care practices, and hierarchical team structures characteristic of healthcare delivery in GCC settings (13). Pretesting with a purposive sample of 30 nursing students (15 regular-track; 15 bridging-track) utilized think-aloud protocols and semi-structured cognitive debriefing interviews to evaluate item comprehensibility, cultural resonance, and response-format suitability, with iterative revisions made until satisfactory clarity was achieved.

#### Psychometric evaluation

*Content validity*. Content validity was evaluated by five subject-matter experts who rated each item’s relevance to the construct of interest using a 4-point ordinal scale. Item-level content validity indices (I-CVI) of ≥0.78 guided item retention decisions. The scale-level average content validity index (S-CVI/Ave = 0.92) indicated excellent content validity, consistent with recommended benchmarks.

*Construct validity*. Construct validity was assessed in two sequential phases. In the pilot phase (*n* = 30), exploratory factor analysis (EFA) was conducted using principal axis factoring with Promax oblique rotation, appropriate given the anticipated intercorrelations among factors. Factor retention was guided by eigenvalues >1, scree plot inspection, and parallel analysis, a combination recommended to minimize over- or under-extraction. Results supported the theoretically anticipated seven-factor structure, accounting for approximately 68% cumulative variance.

In the main study phase (*n* = 260), confirmatory factor analysis (CFA) was conducted using AMOS 26.0 with maximum likelihood estimation to test a seven correlated-factors model. Model fit was evaluated using multiple indices in accordance with contemporary psychometric guidelines: the Comparative Fit Index (CFI = 0.92), Tucker–Lewis Index (TLI = 0.91), Root Mean Square Error of Approximation (RMSEA = 0.065; 90% CI [0.058, 0.072]), and Standardized Root Mean Square Residual (SRMR = 0.074). Collectively, these indices indicated acceptable-to-good model fit. All standardized factor loadings were statistically significant (*p* < 0.001) and ranged from 0.52 to 0.84, exceeding the recommended minimum threshold of 0.40.

*Reliability*. Internal consistency was assessed using Cronbach’s alpha (*α*). Subscale-level *α* values ranged from 0.80 to 0.83, and the total-scale *α* was 0.89, indicating good to excellent internal consistency across all dimensions. Corrected item–total correlations ranged from 0.45 to 0.78, and item-deletion diagnostics confirmed that no item removal would improve total-scale reliability, supporting retention of all 38 items in their reverse-coded form.

## Ethical considerations

This study was conducted in full accordance with the ethical principles outlined in the Declaration of Helsinki (2013 revision), the Belmont Report, the Saudi National Committee of Bioethics (NCBE) guidelines, and the institutional policies of Northern Border University (NBU). Ethical approval was granted by NBU’s Local Committee of Bioethics (Reference No.: 8/25/H; HAP-09-A-043; approved 30 January 2025).

Participation was entirely voluntary and uncompensated. Digital informed consent in Arabic was obtained from all participants before questionnaire access; the consent process clearly delineated study objectives, procedures, potential risks and benefits, confidentiality protections, and participant rights, including the right to withdraw without consequence. No personally identifiable data were collected; the survey architecture structurally separated the consent record from response data, ensuring respondent anonymity.

All data were stored on AES-256–encrypted institutional servers, with access secured via multi-factor authentication (MFA) and data transmission protected by SSL/TLS protocols. Data will be retained for 5 years following study completion, after which secure destruction will be implemented in compliance with institutional data governance policy. No third-party data sharing occurred, and all disseminated findings present aggregated, de-identified results exclusively.

## Data collection

An anonymous, mobile-optimized survey was administered via Google Forms and disseminated to the target population through institutional email and official WhatsApp groups moderated by course representatives. All eligible nursing students in the accessible population (*N* = 336; 240 regular and 96 bridging) were invited, and 260 responded, yielding a 77.4% response rate. Recruitment occurred through institutional email with up to three weekly reminders to enhance participation. Participation was voluntary, with the invitation clearly emphasizing anonymity, lack of incentives, and no academic consequences.

The survey landing page provided detailed participant information, including the study purpose, eligibility criteria, procedures, potential risks and benefits, confidentiality measures, and researcher contact details. To reduce missing data, forced-response logic was applied to the consent item and key scale items. A 7-day completion window accommodated students’ schedules, and browser/device safeguards prevented duplicate submissions. Three systematic reminder notifications were issued to non-respondents at weekly intervals.

## Results

The study included 260 nursing students who met the eligibility criteria and provided complete responses. Table 1 presents the sociodemographic and academic characteristics of participants. The majority of students (68.5%, *n* = 178) were aged 20–29 years, while 30.0% (*n* = 78) were 30 years or older, and 1.5% (*n* = 4) were younger than 20 years. Female students comprised 67.7% (*n* = 176) of the sample, with male students accounting for 32.3% (*n* = 84). Regarding marital status, 75.8% (*n* = 197) were unmarried, and 24.2% (*n* = 63) were married. Academic level distribution revealed that the majority of participants were third-year students (56.5%, *n* = 147), followed by first-year students (21.5%, *n* = 56), fourth-year students (20.8%, *n* = 54), and second-year students (1.2%, *n* = 3). Program type distribution showed that 70.0% (*n* = 182) were enrolled in the regular undergraduate program, while 30.0% (*n* = 78) were in the bridging program. Academic performance was generally high, with 80.8% (*n* = 210) of students achieving a GPA above 3.5, and 19.2% (*n* = 50) with a GPA of 3.5 or below. This demographic profile reflects a predominantly young, female, academically high-performing sample with substantial representation from both educational pathways.

**Table 1:** Sociodemographic and academic characteristics of participants (*N* = 260).

Characteristic	Category	*n* (%)
Age (years)	<20	4 (1.5)
	20–29	178 (68.5)
	≥30	78 (30.0)
Gender	Male	84 (32.3)
	Female	176 (67.7)
Marital status	Unmarried	197 (75.8)
	Married	63 (24.2)
Academic level	First year	56 (21.5)
	Second year	3 (1.2)
	Third year	147 (56.5)
	Fourth year	54 (20.8)
Program type	Regular	182 (70.0)
	Bridging	78 (30.0)
GPA	≤3.5	50 (19.2)
	>3.5	210 (80.8)

GPA, grade point average.

### Prevalence and patterns of perceived barriers

Table 2 summarizes the mean scores and agreement levels for all 38 items across the seven barrier subscales. Overall, students reported moderate to high levels of agreement with multiple barrier dimensions, indicating substantial challenges in applying physical assessment skills clinically. Within the Reliance on Others and Technology subscale, the highest mean score was observed for the item “Physical assessment is the responsibility of medical or allied health staff” (M = 3.45, SD = 0.95), with 85.4% (*n* = 222) of students agreeing or strongly agreeing, suggesting role confusion regarding professional responsibilities. Additionally, 75.4% (*n* = 196) agreed that they “gather all physical assessment data using electronic monitoring devices” (M = 3.22, SD = 1.01), and 66.2% (*n* = 172) endorsed the belief that “the use of technology reduces the need for nurses’ physical assessment skills” (M = 3.06, SD = 1.16), reflecting technological over-reliance.

**Table 2:** Perceived barriers to physical assessment across seven subscales (*N* = 260).

Barrier item	M ± SD	Agree/strongly agree *n* (%)
Subscale 1: Reliance on Others and Technology		
It’s not the nurse’s role to conduct physical assessment	2.08 ± 1.22	72 (27.7)
Gather all data using electronic monitoring devices	3.22 ± 1.01	196 (75.4)
Technology reduces need for physical assessment skills	3.06 ± 1.16	172 (66.2)
Nurses do not need many physical assessment skills	2.38 ± 1.07	99 (38.1)
Physical assessment is only the doctor’s role	2.22 ± 1.12	82 (31.5)
Relying on monitoring equipment for assessment data	2.97 ± 1.11	170 (65.4)
Physical assessment used only when patient deteriorates	2.08 ± 1.08	67 (25.8)
Physical assessment is medical/allied health staff responsibility	3.45 ± 0.95	222 (85.4)
Subscale 2: Lack of Time and Interruptions		
Task-oriented work prevents physical assessment use	2.78 ± 1.05	158 (60.8)
Lack of time is a barrier	3.73 ± 1.01	228 (87.7)
Lack of time for in-depth physical assessment	3.54 ± 0.99	222 (85.4)
No time due to workload	3.25 ± 1.08	195 (75.0)
Documentation prevents time for physical assessment	3.13 ± 0.99	191 (73.5)
Subscale 3: Ward Culture		
Too many interruptions prevent physical assessment	3.34 ± 1.06	204 (78.5)
Ward culture is a barrier	3.11 ± 1.06	180 (69.2)
Assessment done in limited ways due to ward practices	3.40 ± 0.93	213 (81.9)
Assessments not valued by coworkers	3.07 ± 1.04	179 (68.8)
Ward culture discourages physical assessment	3.08 ± 1.07	183 (70.4)
Subscale 4: Lack of Confidence		
Feel support from colleagues	3.59 ± 1.00	226 (86.9)
Lack confidence in accurate performance	2.73 ± 1.06	148 (56.9)
Worry about ability to correctly use skills	3.09 ± 1.04	186 (71.5)
Lack confidence in deciding which skills to use	2.90 ± 1.05	161 (61.9)
Competently use physical assessment skills	3.77 ± 0.96	238 (91.5)
Subscale 5: Lack of Nursing Role Models		
Skills are role-modeled by experienced nurses	3.75 ± 0.94	235 (90.4)
Nurse leaders promote skill use	3.71 ± 1.00	231 (88.8)
Nurses encourage each other to use skills	3.72 ± 1.03	227 (87.3)
Lack of experienced staff to role model skills	3.57 ± 1.03	220 (84.6)
Subscale 6: Lack of Influence on Patient Care		
Information used to develop plan of care	3.81 ± 0.85	246 (94.6)
Skills make positive difference in patient care	3.85 ± 0.95	238 (91.5)
Skills improve quality of nursing care	3.94 ± 0.95	240 (92.3)
Information used to make treatment decisions	3.79 ± 0.96	236 (90.8)
Subscale 7: Specialty Area		
Skills relevant to specialty area	3.72 ± 0.89	241 (92.7)
Do not use skills outside specialty area	3.20 ± 0.89	208 (80.0)
Specialty determines skills used	3.61 ± 0.88	240 (92.3)
Skills restricted to specialty area	3.10 ± 1.04	186 (71.5)
Skills determined by ward acceptability	3.56 ± 0.93	234 (90.0)

M, mean; SD, standard deviation; Likert scale: 1, strongly disagree; 5, strongly agree.

Regarding the Lack of Time and Interruptions subscale, the item “Lack of time is a barrier to using physical assessment skills” received the highest endorsement (M = 3.73, SD = 1.01), with 87.7% (*n* = 228) agreement, indicating pervasive time constraints. Similarly, 85.4% (*n* = 222) agreed that they “lack time to do an in-depth physical assessment of patients” (M = 3.54, SD = 0.99), and 75.0% (*n* = 195) reported that workload prevents the use of physical assessment skills (M = 3.25, SD = 1.08). For the Ward Culture subscale, students most strongly endorsed the item “Assessment is done a certain way in the ward which limits the extent of physical assessment” (M = 3.40, SD = 0.93, 81.9% agreement, *n* = 213), followed by “Too many interruptions during work prevent doing a physical assessment” (M = 3.34, SD = 1.06, 78.5% agreement, *n* = 204).

Within the Lack of Confidence subscale, the highest mean score was for “Physical assessment skills need to be done competently by nurses” (M = 3.77, SD = 0.96), with 91.5% (*n* = 238) agreement, reflecting awareness of competency expectations. However, students reported moderate agreement with “Lack of confidence in accurately performing physical assessment skills” (M = 2.73, SD = 1.06, 56.9% agreement, *n* = 148), indicating variability in self-perceived confidence. The Lack of Nursing Role Models subscale revealed that 90.4% (*n* = 235) agreed that “Physical assessment skills are role-modeled by experienced nurses in the ward” (M = 3.75, SD = 0.94), and 88.8% (*n* = 231) agreed that “Nurse leaders promote the use of physical assessment skills” (M = 3.71, SD = 1.00), suggesting positive role modelling environments, though 84.6% (*n* = 220) also endorsed “There is a lack of experienced nursing staff to role model physical assessment skills” (M = 3.57, SD = 1.03), indicating contradictory perceptions.

For the Lack of Influence on Patient Care subscale, the highest endorsement was for “The ability to use physical assessment skills improves the quality of nursing care” (M = 3.94, SD = 0.95, 92.3% agreement, *n* = 240), followed by “The ability to use physical assessment skills makes a positive difference in patient care” (M = 3.85, SD = 0.95, 91.5% agreement, *n* = 238), demonstrating strong recognition of clinical value. Finally, regarding the Specialty Area subscale, 92.7% (*n* = 241) agreed that “Physical assessment skills are relevant to nurses in the specialty area” (M = 3.72, SD = 0.89), and 92.3% (*n* = 240) agreed that “The specialty area determines the physical assessment skills that nurses use” (M = 3.61, SD = 0.88), indicating awareness of context-specific skill application.

### Differences in perceived barriers by gender and program type

Table 3 presents the results of Mann–Whitney U tests comparing median barrier scores across gender and program type. Significant gender differences emerged across multiple subscales. Female students reported significantly higher median scores for Reliance on Others and Technology (median = 3.50) compared to male students (median = 3.13, *Z* = −4.216, *p* < 0.001), indicating greater perceived technological over-dependence among females. Statistically significant gender differences were also observed for Ward Culture (*Z* = −2.235, *p* = 0.025), Lack of Confidence (*Z* = −2.274, *p* = 0.023), and Specialty Area (*Z* = −3.647, *p* < 0.001), with females consistently reporting higher barrier perceptions. The total barrier score demonstrated significant gender differences (*Z* = −4.006, *p* < 0.001), with female participants reporting higher overall median scores (median = 3.25) compared to males (median = 3.00).

**Table 3:** Differences in perceived barriers by gender and program type (*N* = 260).

Barrier Subscale	Male (median)	Female (median)	Regular (median)	Bridging (median)	Gender		Program type	
					Z	*p*	Z	*p*
Reliance on Others/Technology	3.13	3.5	3.5	3.25	−4.216	<0.001	−2.276	0.023
Lack of Time/Interruptions	2.8	2.8	2.8	2.6	−0.519	0.604	−2.215	0.027
Ward Culture	2.8	2.8	3	2.6	−2.235	0.025	−1.368	0.171
Lack of Confidence	3	3	3.25	3	−2.274	0.023	−1.516	0.13
Lack of Nursing Role Models	3.5	4	4	4	−1.197	0.231	−0.109	0.913
Lack of Influence on Care	3.5	3.5	3.5	3.5	−1.146	0.252	−1.141	0.254
Specialty Area	3	3.2	3	3	−3.647	<0.001	−2.466	0.014
Total Barrier Score	**3**	3.25	3.25	3.09	−4.006	<0.001	−3.139	0.002

Z, standardized test statistic from Mann–Whitney *U*-test; *p*, two-tailed probability value. Bold indicates statistical significance at *p* < 0.05.

Significant differences also emerged between program types. Regular program students reported significantly higher median scores than bridging students for Reliance on Others and Technology (median = 3.50 vs. 3.25, *Z* = −2.276, *p* = 0.023), Lack of Time and Interruptions (median = 2.80 vs. 2.60, *Z* = −2.215, *p* = 0.027), and Specialty Area (median = 3.00 vs. 3.00, *Z* = −2.466, *p* = 0.014). The total barrier score was significantly higher for regular program students (median = 3.25) compared to bridging students (median = 3.09, *Z* = −3.139, *p* = 0.002), suggesting that students with prior clinical experience (bridging cohort) perceive fewer barriers overall. No significant differences were observed between groups for Lack of Nursing Role Models (*p* = 0.231 for gender; *p* = 0.913 for program type) or Lack of Influence on Patient Care (*p* = 0.252 for gender; *p* = 0.254 for program type).

### Predictors of perceived barriers: multiple linear regression analysis

Table 4 presents the results of the multiple linear regression analysis examining demographic predictors of total barrier scores. Prior to analysis, all regression assumptions were systematically verified: linearity was confirmed through scatterplot inspection; independence of residuals was supported by the Durbin–Watson statistic (*d* = 1.89); homoscedasticity was confirmed through structured residual plot examination; multicollinearity was absent across all predictors (all VIF < 2.5, tolerance > 0.40); and normality of residuals was deemed acceptable based on histogram and normal probability–probability (P–P) plot inspection.

**Table 4:** Multiple linear regression analysis predicting total barrier scores (*N* = 260).

Predictor	*B*	*SE*	*β*	*t*	*p*	Bootstrap 95% CI
Constant	2.852	0.096	—	29.773	<0.001	[2.655, 3.049]
Gender (female vs. male)	0.227	0.047	0.279	4.853	<0.001	[0.138, 0.321]
Program type (bridging vs. regular)	−0.474	0.07	−0.571	−6.802	<0.001	[−0.584, −0.360]
Age (years)	0.023	0.004	0.443	5.209	<0.001	[0.016, 0.031]

B, unstandardized regression coefficient; SE, standard error; *β*, standardized regression coefficient. Bootstrap results based on 5,000 resamples using the bias-corrected accelerated (BCa) method. Standardized coefficients (*β*) reflect the relative predictive weight of each variable within this model and should not be interpreted as effect size indices. Model summary: *R*^2^, 0.217, adjusted *R*^2^, 0.207, *F*(3, 256), 23.582, *p* < 0.001.

The overall regression model was statistically significant, *F* (3, 256) = 23.582, *p* < 0.001, with the model accounting for 21.7% of the variance in perceived barriers to physical assessment (*R*^2^ = 0.217, adjusted *R*^2^ = 0.207). While statistically robust, this explanatory magnitude is modest, indicating that the three demographic predictors examined collectively account for approximately one-fifth of the variability in barrier perceptions. The remaining variance (approximately 78%) is attributable to factors not captured within the current model, including, but not limited to, clinical placement experiences, educational environment quality, self-efficacy, and institutional support structures, underscoring the complexity of barrier formation and the need for multifaceted explanatory frameworks in future inquiry. Bootstrap analysis with 5,000 samples was conducted to enhance the robustness of parameter estimates and to provide bias-corrected confidence intervals that account for potential residual assumption violations.

Gender emerged as a statistically significant predictor (*β* = 0.279, *t* = 4.853, *p* < 0.001, 95% CI [0.138, 0.321]), with an unstandardized coefficient of *B* = 0.227, indicating that female students reported meaningfully higher perceived barriers than male students. Within the context of this model, gender demonstrated a statistically reliable but comparatively modest unique contribution to the prediction of total barrier scores relative to the other predictors examined.

Program type was the strongest predictor in the model (*β* = −0.571, *t* = −6.802, *p* < 0.001, 95% CI [−0.584, −0.360]; *B* = −0.474), indicating that bridging-track students reported significantly lower perceived barriers compared with regular-track students. As the largest standardized coefficient in the model, this finding reflects that program type accounted for the greatest unique proportion of explained variance among the three demographic predictors examined. This pattern suggests that prior clinical exposure and professional socialization associated with bridging programs may substantively attenuate barrier perceptions, a relationship warranting further examination in longitudinal and experimental designs.

Age demonstrated a statistically significant positive association with perceived barriers (*β* = 0.443, *t* = 5.209, *p* < 0.001, 95% CI [0.016, 0.031]; *B* = 0.023), indicating that older students tended to perceive greater barriers to physical assessment. Age represented the second-strongest predictor in the model by standardized coefficient magnitude, although the unstandardized coefficient (*B* = 0.023) indicates that each additional year of age was associated with a small absolute increment in total barrier score, signaling a statistically detectable but contextually nuanced relationship. All bootstrap confidence intervals excluded zero, confirming the statistical stability and replicability of these estimates across resampling iterations.

The constant term (*B* = 2.852, *p* < 0.001, 95% CI [2.655, 3.049]) represents the estimated baseline barrier score when all predictors are held at their reference levels. Taken together, these findings indicate that demographic characteristics, particularly program type, age, and gender, are statistically significant, albeit modest, contributors to nursing students’ perceptions of barriers to physical assessment. The limited explanatory power of the model highlights that demographic factors alone provide an incomplete account of barrier formation, and that future studies should incorporate psychosocial, organizational, and curricular variables to model the antecedents of perceived barriers more comprehensively.

## Discussion

Physical assessment is a foundational nursing competence that supports early detection of patient deterioration and clinical decision-making (3). In this study at Northern Border University, nursing students reported moderate-to-high perceived barriers to acquiring and applying physical assessment skills in clinical settings. The pattern suggests barriers are driven less by perceived irrelevance and more by role expectations, clinical workflow constraints, and variability in clinical teaching, findings consistent with international evidence on theory–practice gap in assessment performance (5, 19). Interpreted through social cognitive theory (22), the results indicate that students’ behaviors are shaped by reciprocal influences among personal beliefs (e.g., confidence), environmental conditions (e.g., ward norms), and reinforcement opportunities during placements.

A central barrier was students’ belief that physical assessment is primarily the responsibility of medical, reflecting role ambiguity that can restrict nursing autonomy and reduce students’ expectations that bedside assessment is a core nursing responsibility (2, 11). When assessment is viewed as someone else’s remit, students may perform fewer complete examinations, limiting mastery experiences required for skill development and confidence growth (22). This aligns with prior work showing that unclear professional boundaries reduce physical assessment use in clinical practice (5).

Students also reported substantial reliance on electronic monitoring devices believing that technology reduces the need for nurses’ physical assessment skills. While technology can enhance efficiency, heavy dependence may reduce hands-on examination and contribute to skills not being practiced consistently, particularly in environments where monitoring is perceived as a sufficient “safety net” (5, 7). This is clinically relevant because physical assessment involves observation, palpation, percussion, auscultation, and interpretive reasoning – competencies that require repeated performance in authentic contexts rather than passive data review (3).

Time-related barriers were among the most strongly endorsed. Students reported a lack of time for in-depth assessment, workload pressures, and interruptions as key constraints. These findings mirror evidence that competing clinical demands limit comprehensive assessment even when nurses and students recognize its value (8, 23). Students also perceived that assessment is performed “a certain way” on the ward, suggesting that local routines and implicit norms shape what is considered feasible or expected. Such ward-level practices can reinforce abbreviated assessment behaviors and amplify the theory–practice gap described in nursing education research (10, 19, 28).

From a social cognitive theory perspective, these results reflect strong environmental constraints: when the clinical setting signals that speed and task completion take precedence, students may deprioritize comprehensive assessment despite positive outcome expectations (22, 29). Over time, this may normalize minimal assessment practices and reduce opportunities for feedback, correction, and improvement.

Students strongly agreed that physical assessment should be performed competently by nurses, yet they reported only moderate agreement regarding confidence in accurately performing these skills. This discrepancy suggests awareness of professional standards but variable readiness to enact them consistently during placements. Similar patterns have been reported when assessment skills are taught in curricula but not reliably reinforced in clinical rotations (5, 20).

Perceptions of role modelling were mixed. Students indicated that experienced nurse’s role-model assessment and that leaders promote its use, but they also agreed that there is a lack of experienced staff available to do so. This may reflect uneven exposure across different units, shifts, and preceptors, producing inconsistent observational learning opportunities. Because vicarious learning is important for skill uptake, this variability may contribute to uneven confidence and inconsistent application of assessment skills (22, 24, 30).

Students strongly agreed that physical assessment improves nursing care quality—an important interpretive point: barriers occurred despite high perceived clinical value. Interventions should focus on increasing feasibility and practice opportunities rather than persuading students of relevance (5, 19). Students also recognized that specialty context shapes which skills are used, consistent with evidence that assessment practice is shaped by patient acuity and unit expectations (4).

Group comparisons showed that female students reported higher perceived barriers than male students (e.g., total barrier score, *p* < 0.001). In the Saudi context, cultural norms and mixed-gender interaction expectations may influence comfort and confidence during certain assessment situations, potentially limiting practice opportunities (9, 13, 27). Program type differences were especially notable. Regular-track students reported higher barriers than bridging-track students, with program type being the strongest predictor in the regression model (*p* < 0.001). This plausibly reflects benefits of prior clinical exposure in the bridging cohort, which may increase self-efficacy and reduce perceived disruption from ward culture and time constraints (24, 25). Age was also a significant positive predictor (*p* < 0.001), suggesting that older students may face additional constraints [e.g., responsibilities affecting practice time (18, 26)].

The regression model explained modest variance (*R*^2^ = 0.217), indicating that demographic factors are meaningful but insufficient to explain most variability in barrier perceptions. This supports broader models that incorporate organizational and instructional variables, such as placement quality, preceptor practices, and learning support (5, 19).

## Implications for education and clinical training

These findings support targeted, feasible approaches to reduce barriers. First, curricula and clinical partners should explicitly reinforce physical assessment as a nursing responsibility and clarify expectations during placements (3, 11). Second, competency-based practice supported by simulation can increase skill repetition and feedback in a controlled environment, strengthening mastery experiences and confidence (16). Third, clinical teaching should prioritize consistent role modelling and coaching by prepared preceptors to reduce unit-to-unit variability in observational learning (8, 10). Finally, given national workforce goals, aligning educational outcomes with clinical expectations may strengthen nursing capability and support broader system priorities in Saudi Arabia (15, 17).

## Limitations and future directions

While this study provides valuable insights into the barriers affecting physical assessment skill acquisition, its findings must be interpreted in the context of several important limitations. First our reliance on self-reported data for measuring perceived barriers introduces susceptibility to social desirability bias, this means that participants may have provided answers they thought were more positive or consistent with academic expectations. As a result, some sensitive barriers, like a lack of confidence or perceived ambiguity in professional roles, may be underestimated. Second, objective performance measures to support the self-reported data were not included in the study. A more thorough and reliable assessment of students’ actual skill proficiency and its relationship to their perceived barriers would have been possible by using direct observation techniques like Objective Structured Clinical Examinations (OSCEs). Third, while our regression model was statistically significant, its modest explanatory power (*R*^2^ = 21.7%) indicates that a substantial portion of the variance in perceived barriers remains unexplained by the predictors included in our model (age, gender, and program type). This suggests that other unmeasured variables, such as individual learning styles, prior clinical experiences, or specific faculty-student interactions, likely play a significant role. Fourth, the study was conducted within a single-institution context. Although we employed a robust internal probability sampling strategy to ensure representativeness within our university, the findings may not be generalizable to other nursing programs across Saudi Arabia or internationally, which may have different curricula, student demographics, and clinical training environments. Finally, the cross-sectional design of the study captures a single point in time and does not allow for the examination of causal relationships or how perceived barriers may evolve as students’ progress through their academic and clinical training. Future Directions: To overcome these limitations, future studies should use longitudinal, multi-center designs to improve generalisability and monitor how these barriers change over time. A deeper, more complex understanding of the intricate relationship between perception and practice would be possible by incorporating mixed methods approaches, which include qualitative techniques like ethnographic observations and objective skill assessments (like OSCEs). Furthermore, in order to create more thorough explanatory models of the obstacles to learning physical assessment skills, future research should investigate a larger range of potential predictors.

## Conclusion

This study comprehensively illustrates the complex web of barriers hindering physical assessment skill acquisition among Saudi nursing students, shaped by cultural norms, educational gaps, and institutional hierarchies. By addressing these challenges through targeted curricular reforms, gender-sensitive training, and policy advocacy, nursing education can empower students to transcend traditional role constraints and emerge as confident, competent, and indispensable clinicians. The path forward demands not only pedagogical innovation but also a cultural reimagining of nursing’s diagnostic potential in modern healthcare, ensuring that nurses are fully equipped to contribute to high-quality patient care in line with national health objectives.

## Data Availability

The original contributions presented in the study are included in the article/supplementary material, further inquiries can be directed to the corresponding author.
